# Supplementary material for: Patient use of pulse oximetry to support management of COVID-19 in Greater Manchester: A non-randomised evaluation using a target trial approach
Source: PLoS One. 2024 Nov 15;19(11):e0310822. doi: 10.1371/journal.pone.0310822 (PMC11567555; doi:10.1371/journal.pone.0310822)
Supplement: Attachment — Submitted filename: response.docx [file pone.0310822.s003.docx]

Thank you for reviewing our work. Point by point responses are provided below, while we also provide a manuscript document with tracked changes.

Felix Bongomin, MB ChB, MSc, MMed, FECMM
Academic Editor
PLOS ONE

Journal Requirements:

When submitting your revision, we need you to address these additional requirements.

1. Please ensure that your manuscript meets PLOS ONE's style requirements, including those for file naming. The PLOS ONE style templates can be found at

<https://journals.plos.org/plosone/s/file?id=wjVg/PLOSOne_formatting_sample_main_body.pdf> and

<https://journals.plos.org/plosone/s/file?id=ba62/PLOSOne_formatting_sample_title_authors_affiliations.pdf>

**Response:** Done

2. Thank you for stating the following in the Competing Interests section:

"RW, JD, PB, FR and EK were supported by the NIHR Applied Research Collaboration Greater Manchester (NIHR200174) and RW and JD by the NIHR Manchester Biomedical Research Centre (NIHR203308)."

Please confirm that this does not alter your adherence to all PLOS ONE policies on sharing data and materials, by including the following statement: ""This does not alter our adherence to  PLOS ONE policies on sharing data and materials.” (as detailed online in our guide for authors http://journals.plos.org/plosone/s/competing-interests).  If there are restrictions on sharing of data and/or materials, please state these. Please note that we cannot proceed with consideration of your article until this information has been declared.

Please include your updated Competing Interests statement in your cover letter; we will change the online submission form on your behalf.

**Response:**

Previously we stated that “GMCR data has been made available in de-identified format for research but only in a secure environment. Research protocols (and other data requests) must be approved by GMCR and we are unable to make data available outside that process. https://healthinnovationmanchester.com/gms-secure-data-environment-sde-for-health-and-care/ “, which we assumed explains why we cannot share the data. We do not see the competing interests section as relevant to the data availability statement, nevertheless we now have added this to the competing interests statement “As explained in the data availability section, there are restrictions using patient level data and we cannot freely share the data used in this paper through the journal.”

3. Please include your full ethics statement in the ‘Methods’ section of your manuscript file. In your statement, please include the full name of the IRB or ethics committee who approved or waived your study, as well as whether or not you obtained informed written or verbal consent. If consent was waived for your study, please include this information in your statement as well.

**Response:**

Thank you, we have added the statement in the methods section, and we have expanded to cover the points raised:

“This study is based on data from the Greater Manchester Care Record (GMCR), using anonymised, routinely collected data provided in a de-identified format for research. Informed written consent is needed for primary care patient data to be collected for service improvement and research, before data extraction to the GMCR. The study was approved under protocol GMCR RQ-048, on 12/05/2022. As indicated by the University of Manchester ethics decision tool, formal ethical approval was not required for this study.”

4. Please ensure that you refer to Figure 2 in your text as, if accepted, production will need this reference to link the reader to the figure.

**Response:**

Thank you, that was a typo that has now been corrected.

5. We note you have included a table to which you do not refer in the text of your manuscript. Please ensure that you refer to Table 3 in your text; if accepted, production will need this reference to link the reader to the Table.

**Response:**

Thank you, we have corrected the reference so it is easier to find, as per the guidelines.

6. Please include captions for your Supporting Information files at the end of your manuscript, and update any in-text citations to match accordingly. Please see our Supporting Information guidelines for more information: http://journals.plos.org/plosone/s/supporting-information.

**Response:**

Thank you, we have added the information on the 3 supporting information files as per the guidelines.

5. Review Comments to the Author

Reviewer #1: Excellent manuscript. Congratulations on a hard work well presented. One detail that needs clarification: Was the same device used at home for all patients? Same model and technology for pulse oximetry?

**Response:**

Thank you for your kind assessment. The pulse oximeters were standardised and provided via the NHSE central team and ordered in bulk for use with the intervention. The exact model was the OxyWatch portable non-invasive oximeter, by ChoiceMMed [OxyWatch Pulse Oximeter | Win Health Medical Ltd (win-health.com)](https://www.win-health.com/oxywatch-fingertip-pulse-oximeter). We have clarified this now in the manuscript (Study design section).

Reviewer #2: The authors present the results of an observational study assessing the effects of home pulse oximeter monitoring among COVID-19 patients.
Overall, the study is interesting. However several limitations should be addressed:
- Although the authors state that the exposure is the pulse oximeter, the patients who accepted to receive a pulse oximeter, actually seem to participate in a program that facilitated the prompt access to the health care system. That is, a more complex intervention, where pulse oximeters are a component.

**Response:**

The reviewer is correct, and the intervention is not the pulse oximeter itself (a measurement device), rather the information it provides and the resulting actions. We had tried to make this clearer in the original manuscript, but we have now elaborated on this point in the abstract (conclusion) and the discussion (conclusion).

- The demographic and clinical characteristics used to match cases and controls, probably not controlled all potential confounders. No information about body weight is available.

**Response:**

Thank you for raising this point. Of course, unmeasured confounding is a risk in every observational study, and we have now made this clearer in the limitations section – although we do control for a reasonable number of potential confounders. Weight is a concern, and we have highlighted that in the limitation section addition, but it is due to the limited information on this in the clinical records.

- It would be helpful to show information about treatments prescribed (both chronic and for the COVID-19 event), at the time considered as baseline.

**Response:**

This is an interesting suggestion and there are a few possible interpretations, so we will try to cover all of them. If the reviewer implies using medication data to quantify condition severity / health status and include the information in the regression models, for chronic conditions, it is difficult to ascertain severity from health records, although some work has been conducted in this space (from our team as well). Even then, medications are not often used since this results in a “feedback loop”, with more severe patients being prescribed more due to being prescribed more. For the COVID-19 event, it is possible that some patients who were admitted to hospital and required oxygen were prescribed Dexamethasone and/or were part of the RECOVERY trial in 2021. However, by definition, the patients admitted to the CO@H service were not on oxygen and were considered low risk, thus it can be assumed that the vast majority of patients did not have access to COVID19 specific treatments. However, as we don’t have prescribing data, we cannot confirm this (we have expanded the limitations section to cover this). If the reviewer means to report these as descriptive in a table, this would have been potentially interesting (although difficult to report succinctly) but not central to our research question and the validity of our findings. Unfortunately, we did not request for such data because they are so heterogeneous (with thousands of different medications and formulations) and presenting them descriptively was not essential to our analyses.

- It would be informative to show the adjusted survival curves for binary outcomes**.**

**Response:**

This is a good suggestion and we now provide the adjusted survival curves. This are now reported as Figures 3 and 4.

- The limitations of the study, including those of the methods used for causal inferences, should be discussed in a more detailed way.

**Response:**

Thank you, we have expanded on this.
